# Cellular dynamics of myogenic cell migration: molecular mechanisms and implications for skeletal muscle cell therapies

**DOI:** 10.15252/emmm.202012357

**Published:** 2020-11-19

**Authors:** SungWoo Choi, Giulia Ferrari, Francesco Saverio Tedesco

**Affiliations:** ^1^ Department of Cell and Developmental Biology University College London London UK; ^2^ The Francis Crick Institute London UK; ^3^ Dubowitz Neuromuscular Centre Great Ormond Street Institute of Child Health University College London London UK

**Keywords:** cell migration, cell therapy, muscle regeneration, muscle stem cells, muscular dystrophy, Musculoskeletal System, Regenerative Medicine

## Abstract

Directional cell migration is a critical process underlying morphogenesis and post‐natal tissue regeneration. During embryonic myogenesis, migration of skeletal myogenic progenitors is essential to generate the anlagen of limbs, diaphragm and tongue, whereas in post‐natal skeletal muscles, migration of muscle satellite (stem) cells towards regions of injury is necessary for repair and regeneration of muscle fibres. Additionally, safe and efficient migration of transplanted cells is critical in cell therapies, both allogeneic and autologous. Although various myogenic cell types have been administered intramuscularly or intravascularly, functional restoration has not been achieved yet in patients with degenerative diseases affecting multiple large muscles. One of the key reasons for this negative outcome is the limited migration of donor cells, which hinders the overall cell engraftment potential. Here, we review mechanisms of myogenic stem/progenitor cell migration during skeletal muscle development and post‐natal regeneration. Furthermore, strategies utilised to improve migratory capacity of myogenic cells are examined in order to identify potential treatments that may be applied to future transplantation protocols.

## Graphical abstract

This comprehensive review discusses our knowledge on dynamics and mechanisms of myogenic progenitor cell migration during morphogenesis and post‐natal tissue regeneration and provides insights on the possible strategies for the development of safe and efficient cell therapies.

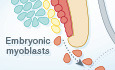

**Glossary**
Amoeboid migration: Amoeboid migration is a type of cell motility (often faster than mesenchymal migration) characterised by cycles of expansion and contraction, which allow cells to squeeze through gaps in the extracellular matrix adopting round or irregular shapes. Leucocytes and cancer cells are two examples of cells which are capable of amoeboid migration
Angiopellosis: An alternative mechanism to leucocyte diapedesis proposed for cells that are not native to the blood circulation to extravasate. During angiopellosis, endothelial cells play a key role in enabling the extravasation of multiple cells during a single event
Cell migration: The process by which translocation of the cell occurs. At the molecular level, cell migration is an orchestrated process and it is performed in a sequential manner
Cell therapy: Treatment strategy based upon delivery of cells as medicine. Can be autologous (i.e. patient's own cells) or allogenic (i.e. from donors); if autologous, cells can be genetically corrected prior to delivery (i.e. gene and cell therapy). The transplanted cells can be stem cells, committed progenitors or differentiated cells. Cells are usually delivered to regenerate a diseased tissue but can also be delivered to kill a specific target (e.g. tumour)
Endomysium: Thin layer of connective tissue ensheathing each individual skeletal muscle fibre
Extracellular matrix: Three‐dimensional structure that comprises part of interstitial spaces of tissues, derived from secreted macromolecules. The extracellular matrix plays crucial roles in providing biophysical and biochemical cues as well as structural support to nearby cells
Extravasation (transmigration): The process by which (white blood) cells migrate through endothelial cell layers to exit the circulatory system towards inflamed tissues in which they are required
Fibrosis: Excessive accumulation of extracellular matrix components, found upon abnormal wound healing, often resulting in tissue dysfunction
Filopodia: Slim cellular protrusions containing 10–30 actin filaments in parallel arrays, often found at the leading edge of lamellipodia during cell migration
Focal adhesion: Relatively stable sites of interaction between the cell and the surrounding extracellular matrix. Focal adhesions are multiprotein assemblies essential for functions such as generation of tension/traction forces for cell migration and mechanotransduction
Lamellipodia: Broad, “fan‐shaped”, actin‐based, protrusions generated at the leading edge of cells undergoing mesenchymal migration
Mesenchymal migration: A mode of motility in which polarisation of the cell results in generation of actin‐based structures such as lamellipodia. This allows the formation of adhesions generating traction forces. Actomyosin contractions at the rear of the cell subsequently propel the cell in a directional manner
Mesoangioblasts: *In vitro* progeny of perivascular cells able to give rise to blood vessel lineages (mostly smooth muscle) and mesodermal lineages of the surrounding tissue. Skeletal muscle pericyte‐derived mesoangioblasts have been delivered intra‐arterially in pre‐clinical animal models of muscle diseases and in patients with Duchenne muscular dystrophy
Muscular dystrophy: A heterogeneous group of primary genetic diseases of skeletal muscle, characterised by progressive muscle degeneration, wasting and premature death in the most severe forms
Myoblasts (adult skeletal myoblasts): Committed progeny of satellite stem cells most of which expand and fuse with nearby muscle fibres
Pseudopodia: Temporary cytoplasmic processes of eukaryotic cells
Sarcopenia: A pathological condition characterised by age‐related loss of skeletal muscle mass, strength and function
Satellite cell: Skeletal muscle stem cells (also known as MuSCs) residing between the myofibre's plasmalemma and the surrounding endomysium. Upon activation, satellite cells give rise to committed progenitors called myoblasts (see above) most of which fuse with surrounding fibres (for repair or regeneration); a minority return to quiescence to replenish the self‐renewing stem cell pool of satellite cells
Somite: Sphere of paraxial mesoderm paired bilaterally along the neural tube during embryonic development. Somites give rise to cells which in turn will generate different mesodermal derivatives such as cartilage, bone, muscle and tendons

## Introduction

Cell migration is a fundamental process for embryogenesis, repair and regeneration of skeletal muscle, the most abundant human tissue. During development, skeletal myogenic progenitors migrate towards prospective skeletal muscles of the trunk and limbs, where they also give rise to stem cells responsible for post‐natal repair and regeneration of skeletal muscles: the muscle satellite cells (MuSCs). MuSCs represent the key skeletal muscle stem cell population, residing between the sarcolemma and the endomysium of muscle fibres (Mauro, 1961). Upon activation, MuSCs give rise to committed proliferating progenitors termed myoblasts, which migrate and fuse either amongst themselves or with pre‐existing myofibres to (re)generate and repair skeletal muscle (Watt *et al*, 1987; Morgan *et al*, 1987; Phillips *et al*, 1990; Siegel *et al*, 2009; Ishido & Kasuga, 2011; Baghdadi *et al*, 2018). Although processes that drive activation, proliferation and differentiation of muscle stem cells are well‐studied, the molecular mechanisms responsible for the migratory properties of myogenic cells have not been the focus of extensive investigation.

Migration has implications in cell therapies of muscle diseases such as muscular dystrophies, heterogenous myopathies characterised by progressive muscle wasting (Mercuri *et al*, 2019). Duchenne muscular dystrophy (DMD), the most common form of paediatric muscular dystrophy occurring in 1/3,500–5,000 boys, is caused by mutations in the *DMD* gene encoding dystrophin (Hoffman *et al*, 1987). Dystrophin functions as a shock absorber to stabilise the sarcolemma and *DMD* mutations lead to contraction‐induced degeneration of skeletal myofibres and impaired muscle function (Muntoni *et al*, 2003). DMD patients experience early loss of ambulation and mortality as a result of cardiorespiratory complications, often within the first three decades of life (Mercuri *et al*, 2019). Standardised pharmacological interventions for DMD include administration of corticosteroids (Matthews *et al*, 2016) and in some cases mutation‐specific drugs such as Ataluren and Eteplirsen which have recently been approved in Europe and the USA, respectively (Mendell *et al*, 2013; McDonald *et al*, 2017). Several experimental therapeutic strategies have been investigated for DMD and other muscular dystrophies, including gene therapy and stem cell transplantation (Benedetti *et al*, 2013) .

MuSCs possess extensive self‐renewal capacity and efficiently engraft into mouse muscles upon transplantation (Sacco *et al*, 2008). Therefore, a promising strategy to restore dystrophin expression is cell therapy: a procedure based upon transplantation of healthy donor or autologous (genetically corrected) cells which then fuse with existing multinucleated myofibres or form new fibres, re‐establishing tissue function. Cell‐based approaches to treat DMD have been explored since the 1980s, when transplantation studies of healthy donor myoblasts in the muscles of *mdx* mice, a mouse model of DMD, displayed robust engraftment and rescue of dystrophin expression (Partridge *et al*, 1989). However, the subsequent early phase clinical trials in DMD patients exhibited limited dystrophin restoration and functional amelioration (reviewed in (Tedesco *et al*, 2010)). Major efforts have since been made to circumvent some of the main issues associated with muscle cell therapy such as the host immune response, poor cell survival and limited cell migration post‐injection (Gussoni *et al*, 1992; Karpati *et al*, 1993; Vilquin *et al*, 1994; Bouchentouf *et al*, 2007). Myoblast transplantations were carried out under immunosuppressive regimens (Vilquin *et al*, 1994), and protocols entailing high‐density injections were also implemented (Skuk *et al*, 2007); nonetheless, results remain suboptimal (Skuk & Tremblay, 2015).

Efforts have since been made to overcome the loss of self‐renewal and engraftment potential during MuSC *in vitro* expansion, leading to protocols capable of preserving their regenerative capacity *ex vivo* (i.e. via pharmacological modulation or by application of biomimetic platforms; reviewed in (Judson & Rossi, 2020)). However, the field still looks for highly migratory myogenic cells or methods that mediate improved cell dispersal and dissemination *in vivo*, thereby facilitating the development of treatments for degenerative diseases affecting multiple large muscles or for severe volumetric muscle loss.

There are two predominant methods of cell transplantation into skeletal muscles: the intramuscular route and the systemic/intravascular route. Both modalities are constrained by insufficient migration of donor cells which limits the efficacy of treatments. Intramuscular injections have mostly been performed with skeletal myoblasts. A common observation of this mode of delivery is the formation of chimeric myofibres limited to the trajectory of injection, as opposed to dispersal throughout muscle tissue. This issue limits intramuscular administration to highly localised myopathies such as oculopharyngeal muscular dystrophy (OPMD), for which clinical improvement has been achieved (Périé *et al*, 2014). On the other hand, intra‐arterial delivery of donor cells into major arteries can simultaneously target multiple muscle groups downstream of the injection site and may be better suited for muscular dystrophies with widespread muscle involvement, particularly when affecting skeletal muscles otherwise difficult to access (e.g. diaphragm). However, MuSCs do not efficiently cross endothelial cell layers, and therefore, investigations on systemic delivery have largely focused on alternative myogenic cell types. Nonetheless, rare reports describe intra‐arterial infusion of unpurified myoblasts in rats and non‐human primates resulting in the occasional incorporation of some donor cells into host myofibres (Neumeyer *et al*, 1992; Skuk & Tremblay, 2014). Mesoangioblasts, myogenic cells derived from expansion of a subpopulation of skeletal muscle perivascular cells, exhibit a higher migratory capacity than MuSCs towards skeletal muscles upon intra‐arterial delivery and have proved efficacious in animal models of muscular dystrophy (reviewed in (Benedetti *et al*, 2013)) as well as relatively safe in a first‐in‐human clinical trial (Cossu *et al*, 2016). Nonetheless, myogenic differentiation and overall homing/engraftment of human mesoangioblasts require significant enhancement to reach clinical efficacy.

More recently, significant progress has been made to generate skeletal myogenic cells from induced pluripotent stem cells (iPSCs), which could provide an unlimited source of myogenic cells for cell therapy. However, no defined protocols are currently available for the generation of highly migratory human iPSC myogenic derivatives. Although the rapidly expanding iPSC field is now deriving potent myogenic progenitors increasingly comparable to native MuSCs (e.g. (Chal *et al*, 2015)), the latter are not capable of extravasation upon intravascular delivery.

It is, therefore, critical to develop strategies to enhance the migratory capacity of skeletal myogenic cells. To this aim, we review key studies on dynamics and mechanisms of skeletal myogenic cell migration in the embryo, adult and upon transplantation. Approaches previously applied to boost the transplantation efficacy of MuSCs have been reviewed, as well as mechanisms of trans‐endothelial migration such as those utilised by leucocytes (diapedesis) and cancer cells. We believe that understanding these mechanisms will be critical to engineer or derive populations of myogenic progenitors with enhanced migration capacity for efficacious skeletal muscle cell therapies.

## Essential molecular machinery for myogenic cell motility and migration

Directional migration requires integration and coordination of various molecular and mechanical stimuli. The general dynamics of directional migration can be reduced to repetition of four basic steps: (1) generation of cellular protrusions, (2) adhesion to substrate, (3) contraction and (4) retraction of the cell rear (Vicente‐Manzanares *et al*, 2005). Specific mechanisms vary depending on cell type and context: limited substrate adhesion is required for amoeboid‐based migration, and shifts between modes of migration can occur rapidly in 3D environments in response to changes in levels of confinement and adhesion (Lautscham *et al*, 2015; Winkler *et al*, 2019). Additionally, spatiotemporal regulation is crucial for execution of the aforementioned steps in a sequential manner. This role is often mediated by Rho GTPases, which modulate migration in response to biochemical and biophysical cues by reorganising the actin cytoskeleton (Binamé *et al*, 2010; Ridley, 2015). The two primary modalities of migration are classified as mesenchymal or amoeboid migration. Mesenchymal migration of myoblasts has been relatively well‐studied in comparison with amoeboid migration, as the latter is difficult to observe on bidimensional surfaces (Yamada & Sixt, 2019).

### Mesenchymal migration

**Figure 1:**
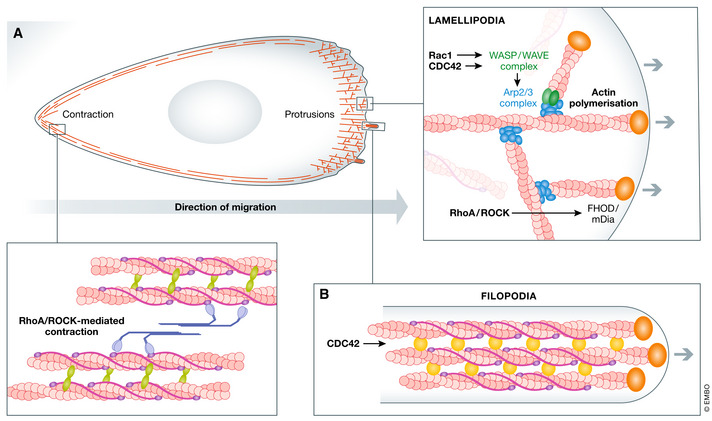
Schematic representation of cytoskeletal elements involved in mesenchymal migration (A) Pathways involved in generation of lamellipodia are outlined. (B) Graphical presentation of a single filopodia, spike‐like protrusions at the leading edge of migration. These structures contain parallel F‐actin bundles crosslinked by fascin (yellow) with polymerisation of actin occurring at the + end of actin filaments, facilitated by diaphanous formins (orange).

Myoblasts display mesenchymal migration on bidimensional monolayers *in vitro* (Kawamura *et al*, 2004). Mesenchymal migration involves generation of protrusions, pseudopodia, at the leading front of the cell, such as the fan‐shaped lamellipodia consisting of branched actin filaments, as well as filopodia comprised of parallel, bundled filamentous actin (F‐actin) (Fletcher & Mullins, 2010). Distinct actin regulators are involved in generation of lamellipodia and filopodia. Lamellipodia formation is primarily dictated by polarised activity of Rho GTPase Rac1 and its downstream effectors such as actin‐related protein 2/3 (Arp2/3) and the WASP‐family verprolin‐homologous protein regulatory complex (WAVE complex) which increase the rate of actin nucleation and subsequently actin polymerisation (Fig 1) (Kawamura *et al*, 2004; Takenawa & Suetsugu, 2007). Filopodia generation, on the other hand, is canonically driven by the Rho GTPase CDC42 with diaphanous formins playing a role in nucleation of actin polymerisation at the migration front (Fig 1) (Mellor, 2010).

Adhesion occurs simultaneously with the extension of cellular protrusions, which are crucial for the generation of traction forces. Binding of integrin receptors (transmembrane heterodimers capable of binding extracellular matrix (ECM) components) facilitates assembly of adhesion complexes which give rise to focal adhesions (FAs) (Harburger & Calderwood, 2009). FAs are multiprotein complexes involved in mechanotransduction as well as cell signalling and are found at the ends of stress fibres. Most FAs possess a laminar arrangement, with integrin cytoplasmic tails, focal adhesion kinase (FAK) and paxillin comprising the signalling module; talin and vinculin making up the intermediate force transduction layer and the innermost actin regulatory layer (Kanchanawong *et al*, 2010). FAs are frequent downstream targets of growth factor‐mediated migration of myogenic cells, and components of FAs are disrupted in some myopathies, resulting in impaired stem cell migration (Leloup *et al*, 2006; Bricceno *et al*, 2014).

Directional migration requires contractile forces for directed cell propulsion. Non‐muscle myosin II‐mediated contractions provide propulsive forces that facilitate directional movement and are crucial for maturation and disassembly of FAs (Schwartz & Horwitz, 2006; Parsons *et al*, 2010). Localisation of contractile activity is facilitated by RhoA, which accumulates at the rear of the cell. RhoA recruits its effector rho‐associated protein kinase (ROCK), capable of myosin light chain phosphorylation, to drive actomyosin contraction at stress fibres. Mechanical forces induced by rear‐end contraction are chiefly responsible for disassembly of FAs and, subsequently, retraction of the rear as the cell body translocates (Crowley & Horwitz, 1995; Chrzanowska‐Wodnicka & Burridge, 1996). Calcium‐dependent proteases such as calpains also play an important role in regulation of adhesion dynamics by cleavage of key FA proteins such as FAK and talin (Chan *et al*, 2010).

### Amoeboid migration of MuSCs

Amoeboid migration involves generation of membrane protrusions (Charras & Paluch, 2008). In contrast to mesenchymal migration, amoeboid migration requires minimal cell‐substrate interactions and can occur under conditions of high confinement (Liu *et al*, 2015). Skeletal muscle injury stimulates activation and proliferation of MuSCs (Yin *et al*, 2013), which subsequently migrate towards sites of injury above the basal lamina (Siegel *et al*, 2009). Although precise mechanisms underlying migration of MuSCs *in vivo* have not been extensively studied, observations of MuSC migration on isolated myofibres indicate employment of an amoeboid mechanism dependent on nitric oxide (NO) and planar cell polarity (PCP) signalling (Otto *et al*, 2011). Other studies with similar experimental approaches indicated the presence of pseudopods on migrating MuSCs (Siegel *et al*, 2009). More recently, live intravital imaging of MuSC migration has similarly shown generation of long protrusions upon activation, suggesting that, *in vivo*, mesenchymal migration is primarily utilised (Baghdadi *et al*, 2018). However, the possibility that MuSCs could interchange between different modalities of migration in a context‐dependent manner remains, although this will require further investigation.

## Mechanisms of skeletal myogenic cell migration during development

**Figure 2:**
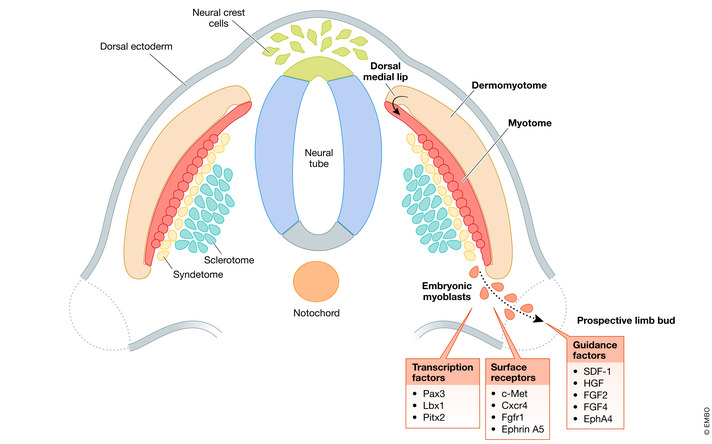
Schematic representation of key transcription factors, surface receptors and guidance factors involved in skeletal myogenic cell migration during development.

During embryonic development, myogenic precursors are required to undergo relatively long‐range migration to give rise to muscles of the developing limbs, tongue and diaphragm (Birchmeier & Brohmann, 2000). Skeletal muscles of the body (trunk and limbs) are derived from the somites: epithelial spheres of compacted paraxial mesoderm which form in pairs alongside the neural tube (Buckingham *et al*, 2003). The paraxial mesoderm segments into somites sequentially in a rostral‐caudal manner and is specified along the dorsoventral axis to form the epithelial dermomyotome dorsally and the mesenchymal sclerotome ventrally, eventually giving rise to cells of the cartilage, connective tissue, muscle, dermis and endothelial lineages (Thorsteinsdottir *et al*, 2011). The dorsomedial lip of the dermomyotome then gives rise to the myotome and subsequently to precursor cells which will generate skeletal myoblasts (Tajbakhsh, 2009). A highly orchestrated migratory event occurs when somitic muscle precursors undergo epithelial‐to‐mesenchymal transition, delaminate and migrate to generate the limb buds, diaphragm and tongue anlagen (Hollway & Currie, 2005; Parada *et al*, 2012; Merrell & Kardon, 2013). These migrating myogenic progenitors are directed by diffusible and cell surface signals, as well as by interactions with the surrounding ECM (Yin *et al*, 2013). As developmental programmes are partially activated during muscle regeneration, understanding migration and homing of myogenic precursors towards muscle anlagen is critical, as these mechanisms may be recapitulated during regeneration or could be exploited for cell transplantation (Fig 2).

Specific transcription factors have been shown to be pivotal for migration of early myogenic precursors, in particular, Ladybird homeobox 1 (Lbx1) and paired box gene 3 (Pax3). Lbx1, a homeobox gene, is expressed in migrating myogenic progenitors and plays a role in directing migratory routes. *Lbx1*‐null mice demonstrated abnormal limb muscles due to defective migration of muscle precursors (Schäfer & Braun, 1999; Brohmann *et al*, 2000). However, muscle precursors lacking *Lbx1* were still able to give rise to the tongue, diaphragm and some limb muscles, indicating that *Lbx1* is necessary for the lateral, but not ventral, migration of embryonic muscle precursors (Gross *et al*, 2000). Another key player is *Pax3*, a homeodomain‐containing transcription factor and an early myogenic cell marker necessary for determining cell fate as well as muscle precursor migration (Williams & Ordahl, 1994; Daston *et al*, 1996; Kassar‐Duchossoy *et al*, 2005; Relaix *et al*, 2005). Several studies performed on Splotch mutant mice, carrying a mutation within the homeodomain of *Pax3*, demonstrated impaired development of limbs due to a loss of the migrating myogenic precursor population, which implies that Pax3 is vital for muscle precursor migration to distal regions of the embryo (Franz *et al*, 1993; Bober *et al*, 1994; Goulding *et al*, 1994; Tajbakhsh *et al*, 1997). Additionally, Splotch mutants display decreased expression of *Lbx1* and *c‐Met*, which encodes the canonical hepatocyte growth factor receptor. It has been suggested that defects of *Lbx1* and *c‐Met* may be due to direct *Pax3*‐mediated regulation of *c‐Met* expression (Epstein *et al*, 1996; Mennerich *et al*, 1998). Another homeobox gene, *Pitx2*, is expressed within prospective limb fields and skeletal muscle anlagen throughout all stages of myogenic development (Shih *et al*, 2007). Abrogation of *Pitx2* expression resulted in anomalies of distal forelimbs. This has been attributed to impaired motility arising from defects in focal adhesions, as numerous regulators of the actin and microtubule cytoskeleton, as well as FA components, displayed changes in expression upon perturbation of *Pitx2* (Campbell *et al*, 2012).

Certain chemokines and their respective receptors are also crucial for homing and maintenance of migratory capacity of muscle progenitors during development. Hepatocyte growth factor (HGF) is present in injured muscles and necessary for migration of myogenic precursors towards developing limb buds (Bladt *et al*, 1995; Tatsumi *et al*, 1998; Lee *et al*, 1999; Dietrich *et al*, 1999). Homozygous null mouse mutants for *c‐Met* show abrogation of myogenic precursor migration to limb buds, diaphragm and tongue and are embryonic lethal (Bladt *et al*, 1995; Amano *et al*, 2002). Additionally, knock‐out (KO) of GRB‐2‐associated binding protein 1 (Gab‐1), a HGF receptor adaptor protein, results in a similar phenotype with loss of extensor muscles, reduced flexor muscle migration and significant disorganisation of hindlimb muscles (Sachs *et al*, 2000; Vasyutina *et al*, 2005). HGF‐c‐Met‐mediated limb precursor migration may involve more than one mechanism. Limb myogenic precursor‐specific KO of B‐Raf, a serine/threonine kinase which acts downstream of c‐Met, has been shown to partially mimic the phenotype of *c‐Met‐null* mutants. B‐Raf has been suggested to promote migration of myogenic cells by direct phosphorylation of Pax3 at serine 205 (Shin *et al*, 2016). On the other hand, myogenic migration towards the developing tongue relies more on PI3K signalling and matrix metalloprotease‐9 (MMP9) (Bandow *et al*, 2004). However, myogenic precursors do not migrate towards ectopic sources of HGF within interlimb regions of the avian embryo, indicating that HGF may not necessarily play a role as a chemoattractant despite its expression along the route of delamination of the prospective limb field and branchial arches (Mennerich *et al*, 1998; Dietrich *et al*, 1999; Birchmeier & Brohmann, 2000). The stromal‐derived factor‐1 (SDF‐1) receptor, CXCR4, is also essential for myogenic progenitor migration, as *CXCR4* ^−/−^ mice display lower numbers of muscle precursors migrating towards prospective limb buds (Vasyutina *et al*, 2005). However, loss of CXCR4 alone did not affect development of the tongue (Huang *et al*, 1999). In *CXCR4^−^* ^/^ *^−^* ^/^ *Gab‐1^−^* ^/^ *^−^* double KO mutant mice, in addition to a reduced number of migrating limb precursors, a small fragment of the tongue anlagen was present. Ectopic application of SDF‐1 was sufficient to direct migration of *CXCR4* ^+^/*Pax3^+^* cells but endogenous migration patterns of myogenic precursors do not completely correlate with SDF‐1 expression patterns, suggesting that alternative factors are involved in the specification of migratory routes (Vasyutina *et al*, 2005). Fibroblast growth factors (FGFs) are also crucial in directing limb myogenic precursors. Avian embryonic myoblasts transfected with dominant‐negative isoforms of FGF receptor 1 (FGFR1) were unable to migrate towards the prospective limb (Itoh *et al*, 1996), whereas FGF2 (also known as basic FGF) and 4 have been shown to induce chemokinesis and chemotaxis of mouse embryonic myoblasts (Webb *et al*, 1997). Interestingly, novel microfluidic tools have revealed that within primary human myoblasts, chemokinesis, rather than chemotaxis, appears to be the main effect exerted by FGF2 (Ferreira *et al*, 2015). A candidate for further specification of migratory routes is ephrin A5 and its receptor EphA4. EphA4 is expressed within the *Pax7^+^* population of lateral dermomyotome and ephrin A5 within the ventral dermomyotome. Ectopic application of ephrin A5 led to a reduction of migration towards the proximal limb bud as well as aberrant accumulation of muscle progenitors within the lateral dermomyotome indicating a role for Ephs and ephrins in migration during limb bud formation (Swartz *et al*, 2001; Stark *et al*, 2011).

## Post‐natal migration of myogenic progenitors

The regenerative capacity of adult skeletal muscle can be observed in both acute injuries and (to some extent) in chronic myopathies (Hardy *et al*, 2016). The endogenous regenerative response is mediated primarily by MuSCs (Relaix & Zammit, 2012). Upon injury, MuSCs are activated and undergo a process of asymmetric division to generate committed progenitors and self‐renewing stem cells (reviewed in (Tedesco *et al*, 2010)). Upon activation in post‐natal skeletal muscles, myoblasts emerge above the basal lamina to migrate towards regions in which they are required to differentiate and fuse with damaged myofibres or with other differentiating myoblasts to generate new fibres. Myoblast migration requires precise modulation of the cytoskeleton, with signalling pathways such as PI3K/Akt and MAPK/ERK pathways chiefly responsible for regulating migration *in vitro* (Kowalski *et al*, 2017; González *et al*, 2017). Additionally, FA regulation is involved in muscle regeneration and disease (Bricceno *et al*, 2014). Several approaches have been explored to enhance the migratory capacity of myogenic cells upon intramuscular transplantation, most of which involve activation of specific signalling pathways and modulation of cell–ECM interactions.

### Signalling pathways in adult myoblast migration

SDF‐1 drives an important signalling pathway in adult myoblast migration. Treating myoblasts with SDF‐1 leads to upregulation of the Rho GTPases CDC42 and Rac‐1, followed by formation of stress fibres and filopodia, whereas silencing of its receptor *Cxcr4*, but not *Cxcr7*, did not increase Rho GTPase expression or cell migration, suggesting direct actin regulation by the SDF‐1/Cxcr4 axis (Kowalski *et al*, 2017). Furthermore, SDF‐1 was shown to regulate the expression of several migration‐associated transcripts, including MMP9, α‐actinin and CAPSN1 (Kowalski *et al*, 2017).

Many other studies assessing migration of myogenic progenitors indicate that promotion of motility occurs frequently via upregulation of MAPK/ERK and PI3K/Akt signalling pathways. HGF‐associated migration is stunted when cells are treated with a MAPK inhibitor, and lamellipodia formation, a crucial step for motility on a monolayer, is abrogated upon PI3K inhibition in C2C12 myoblasts (Kawamura *et al*, 2004; Ishido & Kasuga, 2011; González *et al*, 2017). This decrease in migration is associated with downstream disruption of CDC42‐ and Rac‐1‐mediated actin polymerisation via N‐WASP and WAVE2, as LY294002 treatment also disrupts N‐WASP/WAVE2 localisation at the leading edge of lamellipodia (Kawamura *et al*, 2004; Takenawa & Suetsugu, 2007). Moreover, bisperoxovanadium (BpV), an inhibitor of phosphatase and tensin homolog (PTEN) which negatively regulates PI3K signalling, enhanced myoblast migration (Dimchev *et al*, 2013), whereas pharmacological inhibition of PI3K and MEK reduced myoblast migration (Al‐Shanti *et al*, 2011). Platelet lysates have also shown to enhance motility of C2C12 myoblasts, likely mediated by MAPK and PI3K signalling (Ranzato *et al*, 2009).

Although PI3K/Akt and MAPK/ERK signalling pathways are key mediators of myoblast migration, alternative pro‐migratory signalling pathways also exist. C2C12 myoblasts subject to dominant‐negative Ras‐related protein Ral‐A (dnRalA), an alternative pathway to MAPK/ERK downstream of Ras, showed significantly reduced chemotaxis induced by bFGF, IGF‐1 and HGF (Suzuki *et al*, 2000). Additionally, myogenic progenitors derived from *FGF6^−^* ^/^ *^−^* mice displayed decreased migration upon intramuscular injection, similarly to myoblasts expressing dominant‐negative forms of Ras and Ral (Neuhaus *et al*, 2003).

The TGF‐β superfamily represents another important signalling pathway involved in myoblast migration. Although canonical TGF‐β signalling involves the Smad signalling pathway, myogenic progenitor migration driven by members of the TGF‐β superfamily likely occurs in a non‐canonical manner, via MAPK/ERK or PI3K/Akt signalling. KO of Smad4, using *Myf5‐Cre;Smad4^flox/flox^* transgenic mice specifically targeting Smad4 in myogenic progenitors, has no effect on cell migration of myogenic progenitors during tongue morphogenesis (Massagué, 2012; Han *et al*, 2012). TGF‐β1‐mediated migration of myoblasts has been shown to occur via activation of the MAPK/ERK pathway which facilitates retraction of the trailing end of the cell via upregulation of CAPN2, the catalytic subunit of the ubiquitous calcium‐dependent protease m‐calpain, capable of disassembling FAs by proteolysis of FAK and talin (Franco *et al*, 2004; Leloup *et al*, 2007; Chan *et al*, 2010). Furthermore, bone morphogenetic protein 2 (BMP2), which canonically acts via Smad signalling, has also been suggested to act in a non‐canonical manner. BMP2 regulates cortical actin remodelling at the leading edge via PI3K‐mediated activation of PH‐like domain family B member 2 (LL5β), which recruits the actin crosslinker filamin and, subsequently, promotes cell protrusion (Takabayashi *et al*, 2010; Hiepen *et al*, 2014).

Although significant progress has been made in identifying the aforementioned pathways, mechanisms by which these pathways are coordinated in a spatiotemporal manner to sequentially regulate stages of directional migration of myogenic progenitors require further investigation. Additionally, different signalling pathways, such as those driven by Wnt and nitric oxide (NO), may play context‐dependent roles in alternative modes of migration. One such important external determinant of myogenic migration is the influence of the ECM which is discussed in the following section.

### Influence of the extracellular environment on muscle stem/progenitor cell migration

Interactions between cell surface receptors, primarily integrins, with components of the ECM are crucial to generate traction forces for directional propulsion. Furthermore, timely disassembly and turnover of adhesion complexes are a prerequisite for maximal migration velocity, with dysfunctional FA activity implicated in impaired regeneration and disease. As the ECM is a dynamic entity with a tissue‐specific composition that has been suggested to be altered with age and pathology, changes in ECM composition or stiffness in myopathies may negatively affect migratory capacity of myogenic cells.

#### Turnover of FAs in muscle regeneration and pathology

Modulation of FA proteins affects myogenic cell migration. Tensins are FA‐associated proteins capable of binding cytoplasmic tails of integrins in addition to tyrosine‐phosphorylated proteins by their Src homology 2 (SH2) domains (Chen & Lo, 2003; Lo, 2004). *Tensin‐1*‐null mice display delayed skeletal muscle regeneration and inhibition of tensin‐3 reduces migration of MuSCs (Ishii & Lo, 2001; Chen & Lo, 2003; Baghdadi *et al*, 2018). Additionally, overexpression of microRNA‐708 recapitulates the tensin‐3 inhibitory phenotype, with reduction of phosphorylated FA kinase (p‐FAK), suggesting a role for microRNAs in regulating migration by acting on FAs (Baghdadi *et al*, 2018). Conversely, upregulation of FAK and paxillin, as well as increased proportions of p‐FAK and p‐paxillin, has been suggested to enhance migration. Swine myogenic progenitors with high p‐FAK and p‐paxillin have faster wound closure rates (Wang *et al*, 2016). Similarly, overexpression of platelet and endothelial aggregation receptor‐1 (PEAR‐1; involved in aggregation of platelets and neoangiogenesis) in MuSCs increases p‐FAK, p‐paxillin and vinculin expression, via upregulation and interaction with integrin β1 (Vandenbriele *et al*, 2015; Pang *et al*, 2019). Platelet‐rich plasma was also shown to increase spreading and migration of muscle progenitors by upregulation of FAK and paxillin, whereas deprivation of lysine, an essential amino acid for protein synthesis, resulted in decreased p‐FAK, p‐paxillin as well as decreased cell migration (Tsai *et al*, 2017; Jin *et al*, 2019).

Spinal muscular atrophy (SMA), caused by a deficiency of the survival motor neuron‐1 (SMN‐1) protein, is a severe neuromuscular disorder characterised by muscle atrophy and loss of motor function (D'Amico *et al*, 2011); interestingly, SMN‐1‐deficient myoblasts display reduced motility. This has been attributed to abnormal turnover of FAs, as FA‐associated proteins vinculin, talin‐1 and talin‐2 persist for extended periods of time resulting in prolonged adhesion and, subsequently, in a reduction of motility (Bricceno *et al*, 2014). The downstream effector of Rho GTPase, ROCK‐2, may also be necessary for maturation of FAs as ROCK‐2 pharmacological inhibition resulted in an increased number of vinculin‐positive FAs in myoblasts, which correlated with an increase in migratory velocity (Goetsch *et al*, 2014). Muscle biopsies from SMA patients do not show signs of regeneration, and although this is likely to be caused by several other reasons, the aforementioned observations suggest that reduced regeneration may be partially attributed to decreased migratory capacity of endogenous muscle progenitors as a result of abnormal FA activity.

#### Fibrotic microenvironment and muscle cell migration

Various pathological conditions, including muscular dystrophies and age‐related sarcopenia, are associated with fibrosis: an accumulation of ECM which reduces or impedes tissue function and regeneration (Gillies & Lieber, 2011). In muscular dystrophies, endomysial fibrosis is associated with poor motor control, possibly due to alterations in the load‐bearing, biomechanical role of the ECM (Desguerre *et al*, 2009; Gillies & Lieber, 2011). Fibrotic scar tissue has been postulated to be a limiting factor for the migratory capacity of transplanted cells, as reduction of fibrosis in these tissues via MMP‐1,‐2 or ‐9, has shown to be effective at improving cell migration and engraftment (Gargioli *et al*, 2008; Morgan *et al*, 2010; Pan *et al*, 2015).

An alternative method to reduce fibrotic tissue is by targeting pro‐fibrogenic cytokines, which are released as a result of chronic inflammation in response to recurrent muscle degeneration (Zhou & Lu, 2010). Targeting pro‐fibrogenic signalling pathways in fibroblasts has been shown to reduce skeletal and cardiac muscle fibrosis by inhibition of TGF‐β, which induces fibroblast‐mediated fibrogenesis (Bernasconi *et al*, 1995; McGaha *et al*, 2002; Li *et al*, 2004; Andreetta *et al*, 2006; Cohn *et al*, 2007; Taniguti *et al*, 2011). Furthermore, *scid*/*mdx* mice, which lack T and B lymphocytes, have been reported to have reduced TGF‐β1 activity and muscle fibrosis (Farini *et al*, 2007). These treatments, however, have not been systematically integrated into cell transplantation protocols; future preclinical and clinical studies should therefore focus also on reducing the pre‐existing fibrotic scars to enhance engraftment of intramuscularly or systemically delivered cells.

### Modulating migration: intramuscular delivery

Understanding the migratory properties of myogenic progenitors has significant implications for muscle cell therapies, as lack of migration post‐transplantation remains a key issue that limits therapeutic efficacy. Several approaches have been explored to overcome the limited migratory capacity of transplanted myogenic cells, with varying success. The following section will discuss progress and future implications of these findings.

#### Pretreatment or co‐injection of myoblasts with chemokines

An approach to improve the migratory capacity of myogenic cells is by treating them with factors that stimulate activation and migration during development or regeneration. Extracts from crushed muscles have been shown to activate quiescent MuSCs and to stimulate myoblast migration (Bischoff, 1986, 1990; Allen *et al*, 1995; Corti *et al*, 2001). *In vitro* studies performed using growth factor treatment in transwell or wound healing assays identified several factors able to improve migration of myoblasts (*Table* *1*). Studies attempting to understand underlying mechanisms driven by these factors may be useful for discovery of conserved pathways or mechanisms that could be targeted and modified for application in cell therapy protocols.

**Table 1:** Examples of chemokines and their impact on myoblast migratory capacity *in vitro*.

Chemokine	Concentration	‐fold increase in migration	Cell type	Assay	ECM component	Reference
HGF	10 ng/ml	5.43‐; 5‐	Primary myoblasts (rat; human)	Transwell	Fibronectin	Bischoff (1997), González *et al* (2017)
SDF‐1	10 ng/µl	2.5‐	Primary myoblasts (mouse)	Wound healing	Uncoated	Kowalski *et al* (2017)
TGF‐β	5 ng/ml; 20 ng/ml	4.42‐; 0.73‐	Primary myoblasts (rat); C2C12 myoblasts	Transwell; wound healing	Fibronectin; uncoated	Bischoff (1997), Leloup *et al* (2006)
IGF‐1	40 ng/ml; 100 ng/ml	0.66‐; 3.4‐	C2C12 myoblasts; primary myoblast (mouse)	Wound healing; transwell	Uncoated	Leloup *et al* (2006), Yanagiuchi *et al* (2009)
Insulin	15 µg/ml	0.97‐	C2C12 myoblasts	Wound healing	Uncoated	Leloup *et al* (2006)
FGF‐2	1 ng/ml; 10 ng/ml; 10 ngml; 100 ng/ml; 3.8–7.0 ng/ml	N/A; 7.8‐; 6.4‐ ; 3.4‐; N/A	Primary myoblasts (rat; mouse‐embryonic; mouse; mouse; human)	Transwell; Blind well chemotaxis chamber; Chemotaxis chamber; Chemotaxis chamber; Microfluidics device	Fibronectin; Uncoated; Fibronectin; Uncoated; Uncoated;	Bischoff (1997), Webb *et al* (1997), Neuhaus *et al* (2003), Yanagiuchi *et al* (2009), Ferreira *et al* (2015)
FGF‐4	10 ng/ml	6.7‐	Embryonic myoblasts (mouse)	Blind well chemotaxis chamber	Uncoated	Webb *et al* (1997)
FGF6	10 ng/ml	~5‐	Primary myoblasts (mouse)	Chemotaxis chamber	Fibronectin	Neuhaus *et al* (2003)
PDGF‐BB	50 ng/ml	3.3‐	Primary myoblasts (human)	Transwell	Uncoated	Piñol‐Jurado *et al* (2017)
5% Chick embryo extract	N/A	6.7‐	Primary myoblasts (rat)	Transwell	Fibronectin	Bischoff (1997)

In cases where multiple concentrations were assessed, the concentration that facilitated the greatest fold change of migration was taken.

Several factors displayed in Table 1, including HGF and SDF‐1, have been tested *in vivo* by either treating donor cells prior to administration or by co‐injection. HGF injection into the soleus muscle of mice was sufficient to increase MuSC migration velocity (Ishido & Kasuga, 2011), whereas injection of Sdf‐1‐treated myoblasts into Sdf‐1‐treated, injured gastrocnemius muscles led to an increase in donor‐derived fibres in comparison to controls (Kowalski *et al*, 2017).

#### Modulation of MMPs to facilitate cell migration within skeletal muscles

Extensive fibrosis of skeletal muscles negatively impacts on cell transplantation efficacy, as cells are required to travel through dense connective meshwork for effective dispersion. Similar to the concept of pretreatment irradiation in haematopoietic stem cell transplantation, which “clears space” for donor cells, MMPs may be able to play an analogous role by digesting fibrotic tissues.

MMPs have been previously identified to play a significant role for enhancement of myogenic progenitor migration. Pharmacological inhibition of MMP activity decreased myoblast migration *in vivo* (El Fahime *et al*, 2000). The role of specific MMPs in myogenic progenitors has also been studied. MMP13, an interstitial collagenase, displays increased expression in skeletal muscle of *mdx* mice as well as in muscles acutely injured with cardiotoxin. Overexpression of MMP‐13 in C2C12 myoblasts enhanced migration, which was abrogated upon MMP‐13 inhibition (Lei *et al*, 2013). Pretreatment of host muscles with collagenase and MMP‐7, a matrilysin capable of degrading collagen IV, laminin‐1 and fibronectin, was shown to increase cell engraftment in host muscles (Torrente *et al*, 2000). Furthermore, MMP‐14 has been demonstrated to increase invasive capacity of human but not murine myoblasts, suggesting disparities in the relative importance of MMPs between species (Lund *et al*, 2014). Enhanced growth factor‐mediated migration and motility may also be attributed to increased expression and activity of MMPs. The most likely candidates for this role are MMP‐1, ‐2 and ‐9, which are upregulated by IGF‐1, bFGF and TNF‐α (Allen *et al*, 2003; Lafreniere *et al*, 2004). Intramuscular transplantation of C2C12 myoblasts in *scid*/*mdx* mice with concomitant MMP‐1 administration resulted in a 3‐fold increase in cell engraftment (Wang *et al*, 2009). Additionally, a gene therapy approach to induce MMP‐1 expression in C2C12 myoblasts enhanced engraftment in *scid*/*mdx* mice (Pan *et al*, 2015).

Although MMP‐2 and ‐9 are generally upregulated upon growth factor treatment, MMP‐9 upregulation may be more efficacious in combinatorial therapies (Yanagiuchi *et al*, 2009). Tendon fibroblasts expressing MMP‐9 and the angiogenic factor placenta growth factor (PIGF) were able to restore a microvascular network and reduce fibrosis, enhancing cell therapy efficiency in aged dystrophic mice (Gargioli *et al*, 2008). Similarly, C2C12 myoblasts overexpressing MMP‐9 had superior migratory capacity over C2C12 cells overexpressing MMP‐2 (Morgan *et al*, 2010). Overall, these studies indicate that MMP‐9 expression is advantageous for remodelling of fibrotic tissues found in dystrophic muscles into an environment more favourable for cellular motility.

#### Bioscaffolds for intramuscular cell delivery

Biomaterials have been used to improve dispersion and viability of intramuscularly delivered cells. The advantage of biosynthetic scaffolds is that biophysical and biochemical parameters can be adjusted to modulate cell behaviour (Cezar & Mooney, 2015). Bioscaffolds can also be functionalised with pro‐survival or pro‐migratory factors for myoblasts. Implantation of alginate (a biocompatible polysaccharide) seeded with myoblasts, HGF and bFGF into injured mouse tibialis anterior muscles led to an increase in myoblast migration and muscle mass (Hill *et al*, 2006).

A more practical approach may be to substitute the saline solution in traditional intramuscular injection protocols with hydrogels to improve dispersion and viability of donor cells. A recent study performed by replacing saline with a hydrogel comprised of hyaluronan and methylcellulose resulted in increased donor cell dispersion (Davoudi *et al*, 2018). The increase in engraftment area could also be attributed to the bioactivity of hyaluronan, which has been shown to inhibit myogenic differentiation (Elson & Ingwall, 1980): delays in differentiation may facilitate proliferation and migration/dispersion of donor cells prior to fusion or differentiation. Similarly, a semisynthetic polyethylene glycol and fibrinogen hydrogel increased engraftment of intramuscularly transplanted mesoangioblasts in acute and chronic muscle injury mouse models (Fuoco *et al*, 2012). Therefore, cell–matrix interactions can be exploited to generate an environment advantageous for enhanced cell engraftment.

## Trans‐endothelial migration of myogenic progenitors: state‐of‐the‐art and lessons from “professional” trans migrating cells

Viability of systemic delivery rests on the ability of injected cells to migrate across blood vessel walls while maintaining the capacity to disperse through complex ECM to reach damaged myofibres. Although mesoangioblasts and CD133^+^ cells can be systemically delivered and reach skeletal muscles (reviewed in (Benedetti *et al*, 2013)), precise molecular mechanisms underlying their extravasation process have not yet been established. Greater understanding of mechanisms dictating transmigration in cell types in which extravasation occurs normally (e.g. leucocytes) or pathologically (e.g. metastatic cancer cells) could facilitate development of efficient protocols for the intra‐vascular delivery of myogenic cells.

### Leucocyte diapedesis

**Figure 3:**
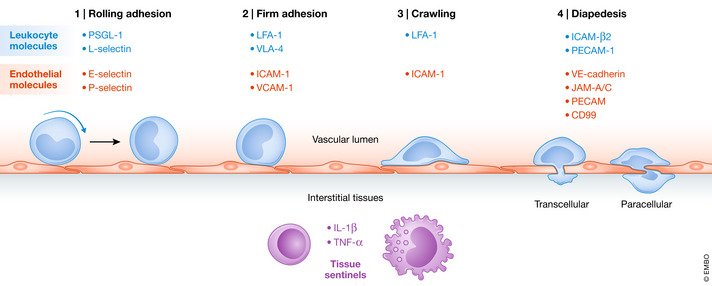
Schematic representation of the sequential events that occur during *leucocyte* diapedesis with key surface molecules involved at each step.

Leucocyte extravasation out of the blood vasculature into target tissues can be summarised into 4 major steps involving different surface protein interactions between leucocytes and endothelium: rolling adhesion, firm adhesion, crawling and diapedesis (Fig 3). As some myogenic progenitors may utilise a similar mechanism when delivered intra‐vascularly (Giannotta *et al*, 2014), targeting components that regulate leucocyte extravasation could improve transmigration of systemically deliverable myogenic cells.

Rolling adhesion requires formation of transient bonds between leucocytes and endothelial cells. Adhesive molecules such as endothelial‐selectin (E‐selectin) and platelet‐selectin (P‐selectin) on endothelium are necessary for interaction with leucocyte surface proteins p‐selectin glycoprotein ligand‐1 (PSGL‐1) and leucocyte‐selectin (L‐selectin) (Kunkel & Ley, 1996; Hickey *et al*, 1999; Stein *et al*, 1999; Zarbock *et al*, 2008). Firm adhesion between leucocytes and endothelial cells requires activation of high affinity integrins on leucocytes. Subsequent generation of dense clusters of intercellular cell adhesion molecule‐1 (ICAM‐1), vascular cell adhesion molecule‐1 (VCAM‐1), lymphocyte function associated‐1 (LFA‐1), very late antigen‐4 (VLA‐4) and galectin‐3 at the leucocyte–endothelium surface, also called focal contacts, enables near‐complete arrest of leucocytes (Dustin & Springer, 1988; Elices *et al*, 1990; Nieminen *et al*, 2008; Muller, 2013). Following arrest, changes in leucocyte morphology occur, involving polarisation and formation of protrusions which promote “crawling” along the surface of endothelial cells (Schnoor *et al*, 2015). Once an exit signal is detected, migration through the endothelial barrier takes place. This can follow either the paracellular or the transcellular route: most transmigration events occur via the paracellular route, through endothelial cell–cell junctions, and require sequential activation of several adhesion proteins. Firstly, ICAM‐β2 integrin interactions are necessary for transient downregulation of vascular endothelial cadherin (VE‐cadherin). This process is pivotal to loosen adherens junctions (Filippi, 2016). Subsequently, sequential interactions of leucocytes with junction adhesion molecule‐A/C (JAM‐A/C), platelet endothelial cell adhesion molecule (PECAM) and CD99 mediate translocation through the intercellular gap (Schenkel *et al*, 2002; Woodfin *et al*, 2009). Alternatively, leucocytes can migrate through the cell body of endothelial cells via transcellular pores, although this is mostly found in regions which necessitate strict regulation of permeability, such as the blood–brain barrier (Lossinsky *et al*, 1989; Wolburg *et al*, 2005). Finally, entry into peripheral tissues requires migration through associated pericyte and perivascular basement membrane layers (Voisin & Nourshargh, 2013).

### Metastatic cancer cells, angiopellosis and other unorthodox extravasation strategies

#### Pathological extravasation: cancer metastasis

Cancer metastasis involves formation of new tumours within tissues and organs distal to the primary mass. This process usually requires cancer cells to intravasate into proximal lymph or blood vessels, circulate, extravasate and, subsequently, proliferate to give rise to secondary tumours (Valastyan & Weinberg, 2011). The efficiency of cancer cell extravasation is a key factor that determines metastatic potential (Leong *et al*, 2014). Extravasation of cancer cells involves the same major steps as leucocytes but displays disparities at the molecular level. Additionally, a key difference between cancer cell and leucocyte extravasation is that cancer extravasation results in disruption of vascular integrity, whereas leucocyte extravasation induces transient, reversible modifications of the endothelium (Strell & Entschladen, 2008). Studying molecules involved in cancer metastasis may reveal general conserved mechanisms necessary for trans‐endothelial migration of non‐leucocyte cells, thus exposing key molecules that may be modulated to enhance the efficiency of myogenic cell trans‐endothelial migration.

Rolling of tumour cells has been shown to require several ligands of E‐ and P‐selectins. Metastases within bone, lymph node and the brain derived from human prostate tumours display increased PSGL‐1 expression, involved in leucocyte rolling (Dimitroff *et al*, 2005). Within PSGL‐1‐negative breast carcinoma cells, CD24 was shown to be responsible for rolling, suggesting the existence of alternative mechanisms for tumour cell rolling (Aigner *et al*, 1998). Isoforms of CD44 may also play crucial roles in cancer cell–endothelium interactions. Knockdown of CD44 glycoform haematopoietic cell E‐/L‐selectin ligand (HCELL) in colon carcinoma cells resulted in reduced binding to HUVECs and increased rolling speed (Burdick *et al*, 2006). CD44 variant (CD44v, a CD44 isoform) expression in colon carcinoma cell lines reduced rolling velocity and increased binding to P‐selectin (Napier *et al*, 2007). Upon knockdown of CD44, the glycoprotein carcinoembryonic antigen (CEA) acts as a compensatory mechanism to mediate colon cancer cell interactions with E‐selectin (Thomas *et al*, 2008). This suggests that although the process of rolling is conserved between tumour cells and leucocytes, disparities exist at the molecular level.

Firm adhesion of cancer cells to the vascular endothelium can occur in a leucocyte‐dependent or ‐independent manner. Tumour cells are capable of recruiting and binding to leucocytes, which subsequently interact with the endothelial cell layer as a proxy (Strell *et al*, 2007; Liang *et al*, 2007). Interestingly, it is currently unknown whether donor myogenic cells are able to interact with leucocytes intravascularly. Tumour cell firm adhesion also takes place independently of leucocytes. Similarly to leucocytes, VLA‐4 acts as the primary VCAM‐1 ligand during tumour cell extravasation. VLA‐4 was shown to be essential for adherence of melanoma cells to VCAM‐1‐expressing endothelial cells, with clones of melanoma cells expressing VLA‐4 displaying increased metastases in IL‐1‐treated mice (Garofalo *et al*, 1995). Additionally, treatment of mammary carcinoma cell lines, possessing high tropism for the brain, with anti‐VLA‐4 antibodies reduced incidence of tumour seeding (Soto *et al*, 2014). Firm adhesion of breast and prostate cancer cells has been shown to involve binding with galectin‐3 on the endothelial surface (Glinsky *et al*, 2000, 2003; Khaldoyanidi *et al*, 2003).

Several molecules involved in cancer cell extravasation and leucocyte extravasation are conserved, but there are also alternative mechanisms of tumour cell extravasation. Transcriptional profiles of circulating tumour cells from 5 different cancer types revealed conserved upregulation of *PECAM‐1*, *JAM3* (JAM‐C) and *F11R* (JAM‐A), which are critical for leucocyte diapedesis (Yadavalli *et al*, 2017). Additionally, melanoma metastases are reduced in mice with an endothelial cell‐specific KO of *JAM3* (Langer *et al*, 2011). Treatment of hepatoma and colon cancer cells with anti‐CXCR4 antibodies had no effect on adhesion but abrogated extravasation and, conversely, pretreatment of these cells with SDF‐1 enhanced extravasation (Gassmann *et al*, 2009). Homophilic CD146 interactions have also been shown to mediate melanoma extravasation, which is decreased upon cell delivery in CD146 KO mice (Jouve *et al*, 2015).

Cancer cell extravasation also involves vasculature modulation. Secretion of c‐terminal fibrinogen‐like domain of angiopoietin‐like 4 (cANGPTL4) by carcinomas and melanomas induces vascular leakiness by interacting with α5β1 integrin, VE‐cadherin and claudin‐5 (Huang *et al*, 2011). Similarly, the glycoprotein osteonectin interacts with VCAM‐1 and facilitates increased vascular permeability, resulting in enhanced melanoma cell extravasation (Tichet *et al*, 2015). Taken, overall this section highlights molecules and mechanisms that might be positively exploited to increase extravasation efficiency of systemically deliverable myogenic cells, although potential detrimental effects on vascular integrity and off‐target extravasation (i.e. into non‐skeletal muscle tissues) will need to be carefully assessed in future studies.

#### Angiopellosis

Recently, an alternative mechanism to diapedesis termed angiopellosis has been proposed for cells that are not native to the blood circulation, including tumour cells (Allen *et al*, 2019). Angiopellosis displays molecular and temporal disparities in comparison to canonical leucocyte extravasation and involves formation of endothelial protrusions which sequester and transport cells into the surrounding parenchyma (Cheng *et al*, 2012; Allen *et al*, 2017). In contrast to leucocyte diapedesis, angiopellosis allows for extravasation of multiple cells during a single event. Knockdown of CD11α, the α‐integrin subunit of LFA‐1 implicated in multiple stages of leucocyte extravasation, abolished diapedesis of leucocytes but not of mesenchymal stromal cells, indicating a difference of surface molecules involved in this process (Allen *et al*, 2017). Overall, angiopellosis appears relevant in the context of muscle cell therapies, as myogenic cells are by definition not native to the circulation. More research on this process, alongside cross‐validation with additional independent studies, will be necessary to exploit this modality of cell migration for the intravascular delivery of myogenic cells.

### Modulating migration: systemic delivery

#### Treating myogenic cells with growth factors, inflammatory chemokines or other small molecules

Cells delivered intra‐arterially must perform two major tasks: (1) transmigration across the endothelial barrier of injured muscle tissues and (2) migration towards regions in which they are required. During embryonic myogenesis, myoblasts in close proximity to endothelial cells undergo a fate transition into pericyte‐like cells (Cappellari *et al*, 2013). This phenomenon can be mimicked *in vitro* by combined application of platelet‐derived growth factor‐BB (PDGF‐BB) (also expressed by regenerating and necrotic myofibres (Piñol‐Jurado *et al*, 2017)) and delta‐like 4 (DLL4; a Notch signalling ligand), inducing pericyte‐like features in both mouse and human myoblasts (Gerli *et al*, 2019). Importantly, treated MuSC‐derived myoblasts displayed enhanced endothelial transmigration capacity *in vitro* and *in vivo* (Gerli *et al*, 2019). Of note, a recent study also showed that PDGF‐BB promotes migration of various types of muscle interstitial cells via interaction with its putative receptor, platelet‐derived growth factor receptor‐β (PDGFR‐β) (Camps *et al*, 2019). As this phenomenon occurs during embryonic development (Cappellari *et al*, 2013), translating this approach to iPSC‐derived myogenic progenitors may result in greater cell transmigration than what observed with adult myoblasts, given the embryonic‐/foetal‐like identity of most currently available skeletal myogenic iPSC derivatives (Xi *et al*, 2020).

Cells delivered intra‐arterially require appropriate exit signals that regulate extravasation. It is therefore not surprising that pro‐inflammatory cytokines secreted by resident cells have previously shown to promote endothelial transmigration. Tumour necrosis factor‐α (TNF‐α), a cytokine secreted by macrophages, natural killer (NK) cells and lymphocytes, enhanced mesoangioblast migration *in vitro* and *in vivo*. Furthermore, highly mobility group box‐1 (HMGB‐1), a pro‐inflammatory cytokine released by necrotic cells or secreted by immune cells, has been shown to promote extravasation and homing of mesoangioblasts (Palumbo *et al*, 2004; Lotze & Tracey, 2005); a similar effect has been reported with nitric oxide (Sciorati *et al*, 2006). Interestingly, other pro‐inflammatory cytokines such as IL‐1, IL‐6 and IL‐10, had no significant effect on donor cell migration (Galvez *et al*, 2006); similarly, lipopolysaccharide (LPS)‐induced inflammation did not stimulate mesoangioblast homing or migration despite upregulation of IL‐1α, IL‐1β and IL‐6 in mouse and rat skeletal muscle (Frost *et al*, 2002; Lang *et al*, 2003; Palumbo *et al*, 2004). Therefore, specific growth factors, chemokines and other ligands can induce pro‐migratory properties to different classes of myogenic cells; translating these protocols to human myogenic cells will be a key step towards their preclinical validation.

#### Modification of cell–endothelial interactions to promote transmigration

Targeting proteins that dictate donor cell–endothelium interactions during extravasation is another promising strategy to promote cell engraftment in dystrophic muscles. Although surface proteins of candidate myogenic cell types differ from those of leucocytes, expression of key molecules involved in leucocyte diapedesis on myogenic cells has been shown to enhance their migration capacity (Tagliafico *et al*, 2004; Galvez *et al*, 2006).

Another strategy for increasing efficiency of transmigration is to target molecules that facilitate sequential migration through paracellular endothelial junctions. KO or inhibition of JAM‐A in mouse models of acute or chronic muscle injury significantly improved engraftment of intra‐arterially delivered mesoangioblasts (Giannotta *et al*, 2014). Downregulation of PW1, a direct transcriptional repressor of JAM‐A, inhibited transmigration of adult mouse mesoangioblasts when delivered via femoral arteries of *scid*/*mdx* mice preventing amelioration of the dystrophic phenotype (Bonfanti *et al*, 2015). Notably, a similar strategy in PECAM‐1‐null mice did not increase cell engraftment, indicating that not all endothelial junction molecules can be targeted to improve cell extravasation (Giannotta *et al*, 2014).

## Future perspectives

Strategies to enhance migration and motility of myogenic progenitors have been investigated in previous studies; however, how these factors regulate the cellular cytoskeleton as downstream output is less studied and may be important to identify key processes or molecular components that may be perturbed in disease or augmented in cell therapies. Additionally, it may be advantageous for future studies to focus on migratory behaviours of myogenic cell types within 3D environments that recapitulate skeletal muscle architecture with higher fidelity with regards to ECM composition, stiffness and multicellular complexity (e.g. Bersini *et al*, 2018; Maffioletti *et al*, 2018). This could be achieved by observing migratory behaviour and cytoskeletal activity in synthetic 3D hydrogels that mimic interstitial ECM or by using decellularised matrices retaining the intrinsic architectural integrity of skeletal muscle tissue (Hughes *et al*, 2010; Webster *et al*, 2016; Yamada & Sixt, 2019). Furthermore, intravital imaging may reveal previously unidentified mechanisms or requirements for optimal migratory capacity of muscle stem cells upon intramuscular or intravascular delivery (Paul *et al*, 2015; Yan *et al*, 2019).

Identifying key genetic programmes and molecular machineries involved in myogenic cell migration could be instrumental to derive or engineer highly migratory cell populations for efficacious muscle cell therapies. For this purpose, several avenues could be explored:

1. Systematic assessment of the migratory properties of interstitial skeletal muscle cells may reveal distinct populations with migration capacity (Tedesco *et al*, 2017). Subsequently, pro‐migratory signals can be modulated to optimise migration and differentiation capacity for maximal engraftment in cell therapies (Gerli *et al*, 2019). Engineering myogenic derivatives from human iPSCs will become increasingly relevant as modifications of current transgene‐based (e.g. (Tedesco *et al*, 2012; Darabi *et al*, 2012)) or transgene‐free protocols (e.g. (Chal *et al*, 2016; Hicks *et al*, 2018)) could generate innovative advanced therapy medicinal products (ATMPs) with controllable proliferation, migration and differentiation capacity.
2. Phenotypic disparities between young and aged myogenic cell types have been investigated (Collins‐Hooper *et al*, 2012; Brown *et al*, 2017; Rotini *et al*, 2018). In addition to regenerative capacity, migration has been suggested to be altered with ageing: studying and understanding migration dynamics in young vs aged myogenic cells may highlight specific pathways to modulate to enhance migration of ATMPs.
3. Lastly, omics‐based comparative studies of myogenic cell populations treated with pro‐migratory factors or small molecules may unravel druggable pathways which could be further modulated to enhance cell motility. For intravascular delivery, similar studies focusing on cells with highly efficient transmigration capacity in health (e.g. leucocytes) or disease (e.g. metastatic cells) could provide insights on strategies to be deployed in next‐generation skeletal muscle cell therapies.

## Author contributions

SWC wrote the manuscript's draft with supervision of GF and FST. FST coordinated the work, contributed to the draft, reviewed and finalised the manuscript and acquired funding.

## Conflict of interest

The authors declare that they have no conflict of interest.

**Pending Issues**

- Elucidating druggable pathways in human myogenic progenitors which can be targeted across different cell populations to enhance their migration without compromising safety, self‐renewal and differentiation potential.
- Development of new models and screening platforms to assess migration potential of ATMPs for muscle diseases upon intravascular delivery (e.g. (Ferreira *et al*, 2015)).
- Development of GMP‐GLP‐defined protocols to enhance migration human transplantable myogenic progenitors, particularly from iPSCs.

**For more information**

- Authors' website: www.tedescolab.org (Twitter: @lab_tedesco)
- Cell migration resources & seminars: https://cellmigration.wixsite.com/seminars (Twitter: @CellMigration)
- Muscle biology resources and seminars: @musclescitalks (Twitter)
- Patients associations (examples):
  - https://www.musculardystrophyuk.org
  - https://dmdhub.org
  - http://www.afm‐telethon.com
  - https://www.actionduchenne.org
  - https://www.mda.org
